# Forced diuresis and dual-phase ^18^F-fluorodeoxyglucose-PET/CT scan for restaging of urinary bladder cancers

**DOI:** 10.4103/0971-3026.59746

**Published:** 2010-02

**Authors:** S Harkirat, SS Anand, MJ Jacob

**Affiliations:** Department of Nuclear Medicine and PET/CT Facility, Army Hospital (R and R), New Delhi, India

**Keywords:** Bladder cancer, FDG-PET/CT, forced diuresis

## Abstract

**Context:**

The results of ^18^F-fluorodeoxyglucose (FDG)-PET imaging carried out with the current standard techniques for assessment of urinary tract cancers have been reported to be less than satisfactory because of the urinary excretion of the tracer.

**Aims:**

To investigate the role of dual-phase FDG-PET/CT in the restaging of invasive cancers of the urinary bladder, with delayed imaging after forced diuresis and oral hydration as the scanning protocol.

**Settings and Design:**

FDG-PET has been considered to be of limited value for the detection of urinary tract cancers because of interference by the FDG excreted in urine. We investigated the efficacy of delayed FDG-PET/CT in the restaging of invasive bladder cancer, with imaging performed after intravenous (IV) administration of a potent diuretic and oral hydration.

**Materials and Methods:**

Twenty-nine patients with invasive cancer of the urinary bladder were included in this study. Patients were divided into two groups: Group I (22 patients) included cases with invasive bladder cancer who had not undergone cystectomy and group II (seven patients) included cases with invasive bladder cancer who had undergone cystectomy and urinary diversion procedure. All patients underwent FDG-PET/CT scan from the skull base to the mid-thighs 60 min after IV injection of 370 mega-Becquerel (MBq) of FDG. Additional delayed images were acquired 60-90 min after IV furosemide and oral hydration. PET/CT data were analyzed as PET and CT images studied separately as well as fused PET/CT images and the findings were recorded. The imaging findings were confirmed by cystoscopy, biopsy or follow-up PET/CT.

**Results:**

The technique was successful in achieving adequate washout of urinary FDG and overcame the problems posed by the excess FDG in the urinary tract. Hypermetabolic lesions could be easily detected by PET and precisely localized to the bladder wall, perivesical region and pelvic lymph nodes. PET/CT delayed images were able to demonstrate 16 intravesical lesions (in 13 patients), with excellent clarity. Lymph node metastases were detected in a total of six patients. Of these, in two patients, FDG-avid lymph nodes were evident only in the delayed images. The information provided by the postdiuretic delayed images changed the PET/CT interpretation in 14 patients of invasive bladder cancer: Recurrent bladder lesions were identified in 12 patients, pelvic lymph node metastasis (only) in one patient and bladder lesion as well as lymph node metastasis in one patient. Distant metastases were detected by PET/CT in two cases. CT scan was false-negative for early recurrence in the bladder wall for seven of 16 lesions. CT also showed two false-positive lesions. There were no false-positives with PET.

**Conclusions:**

Detection of recurrent disease in cases of invasive bladder cancer can be significantly improved by using FDG-PET/CT, with delayed imaging following forced diuresis and oral hydration. Composite PET/CT is superior to CT alone for the restaging of invasive bladder cancers.

## Introduction

Positron emission tomography (PET) has an established role in oncologic imaging and plays an important role in the initial staging, assessment of response to therapy, restaging and detection of recurrence in many types of cancers.[1–8] ^18^F-fluorodeoxyglucose (FDG) is the most commonly used PET tracer in oncologic imaging but has been considered to be of limited value for the detection of urinary tract cancers because of interference by the FDG excreted in urine. A number of techniques have been used to overcome this limitation, especially for evaluation of bladder tumors. The techniques used include complete bladder washout using furosemide injection before image acquisition, retrograde bladder irrigation or postvoid image acquisition. We obtained good results using dual-phase PET/CT scan, before and after administration of a potent diuretic, to delineate recurrent lesions of bladder cancer. We present our findings in this paper.

## Materials and Methods

### Patient population

Twenty-nine consecutive patients with known invasive cancer of the urinary bladder who underwent FDG-PET/CT between April 2006 and March 2008 at our center were included in this retrospective study. All PET/CT scans were requested for restaging or during follow-up after surgery (transurethral tumor resection or cystectomy). The histopathological diagnosis was transitional cell carcinoma (TCC) in all the 29 cases. The cases were divided into two groups: Group I included cases with invasive bladder cancer, who underwent cystectomy (22 patients) and group II included patients with invasive bladder cancer, who had had cystectomy and urinary diversion (seven patients). All bladder-preserved patients underwent cystoscopy and some were also subjected to MRI after PET/CT. The findings of PET, CT scan and PET/CT were correlated with the findings from cystoscopy and biopsy or MRI to confirm or rule out presence of disease. Diagnostic validation was by cystoscopy and biopsy in bladder-preserved patients who had positive PET/CT findings and by radiological (CT/ MRI) correlation/cystoscopic follow-up in PET/CT-negative bladder-preserved cases. In patients with urinary diversion procedures, validation was by radiological correlation and follow-up PET/CT.

### PET/CT protocol and technique

Dual-phase PET/CT scans were carried out in all patients. After the first scan, intravenous (IV) furosemide and oral hydration was given, followed by acquisition of additional delayed images. All the studies were conducted using a dedicated full-ring lutetium orthosilicate (LSO) crystal-based PET/CT scanner (Biograph 2; Siemens Medical Systems, Erlangen, Germany). Nonenhanced CT images were acquired at 130 KV and 90 mA (mean), with a section width of 5 mm. CT-based attenuation corrections were performed for the PET images and reconstruction was carried out using an iterative reconstruction algorithm.

The PET/CT protocol included IV injection of 370 MBq (10 mCi) of FDG after the patient had fasted for 4 h, and confirmation that blood glucose levels were below 140 mg/ dl. Whole-body PET/CT scan (skull base to mid-thighs) was performed 1 h after FDG injection. On completion of the scan, after confirming the hemodynamic stability of the patient, IV furosemide (0.5 mg/kg body weight) was administered. The patients were hydrated orally with 1000-1500 ml water and instructed to evacuate the bladder frequently (at least thrice). Repeat PET/CT scan of the pelvic region was carried out at 60-90 min post furosemide injection (i.e., 150-180 min from the time of the FDG injection). PET/CT images were first analyzed separately as PET and CT images and later with fusion (as composite PET/CT images). PET/CT images before and after furosemide were compared with each other and the findings were correlated with that of cystoscopy and biopsy.

## Results

High tracer activity is usually detected in the urinary tract after standard PET/CT acquisition following FDG administration. Delayed images after furosemide and oral hydration showed marked reduction of activity in the urinary tract. Forced diuresis coupled with oral hydration reduced the urinary tract activity to near-background levels in 21 (95%) of the 22 bladder-preserved patients [Table 1]. Of the seven patients with urinary diversion, reduction in urinary activity to near-background levels was achieved in two patients, while in five patients, significant activity remained in the ileal conduits, although the upper tracts were adequately cleared. Sixteen FDG-avid intravesical lesions (in 13 patients) were visualized with excellent clarity, all being detected only in delayed postdiuretic images [Table 2]. All lesions were found to be hypermetabolic.

**Table 1:** Efficacy of forced diuresis in reducing18F-fl uorodeoxyglucose activity in the bladder

Group	Total no. of patients	No. of patients with adequate washout*	Percentage with adequate washout
I (Bladder preserved)	22	21	95
II (Postcystectomy)	7	5	29

* SUV of bladder urine <2.0 in postdiuretic delayed scan

**Table 2:** PET/CT findings before and after forced diuresis

Location of lesion	PET/CT positive before diuretic (no. of lesions)	PET/CT positive after diuretic (no. of lesions)
Local disease in bladder	0	16
Regional LN	04	06
Distant metastases	02	02

LN - Lymph node

**Figure 1:**
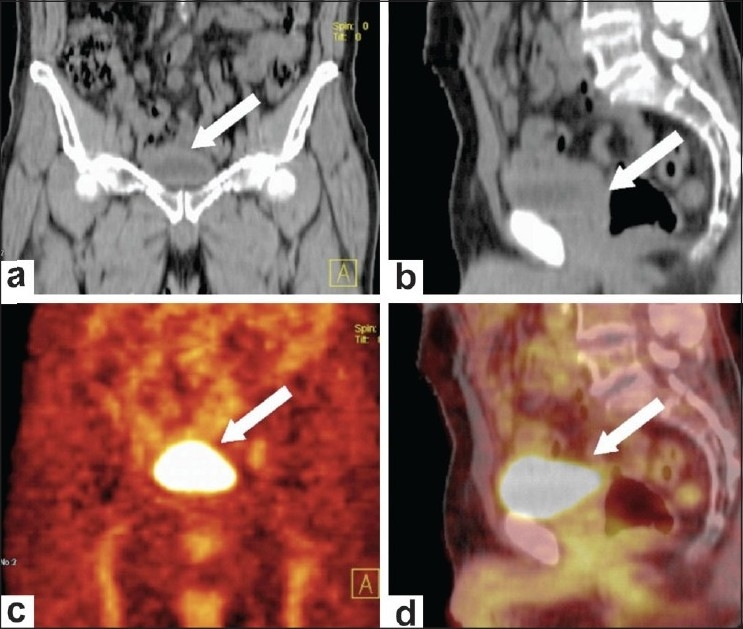
Standard FDG-PET/CT acquisition in a follow-up case of invasive carcinoma of the bladder. Coronal (a) and sagittal (b) plain CT scan images reveal focal bladder wall thickening posteroinferiorly (arrows). Coronal PET (c) and sagittal PET/CT (d) images show a high concentration of tracer in the urine-filled bladder cavity. No wall abnormality is discernable in the bladder (arrows)

**Figure 2:**
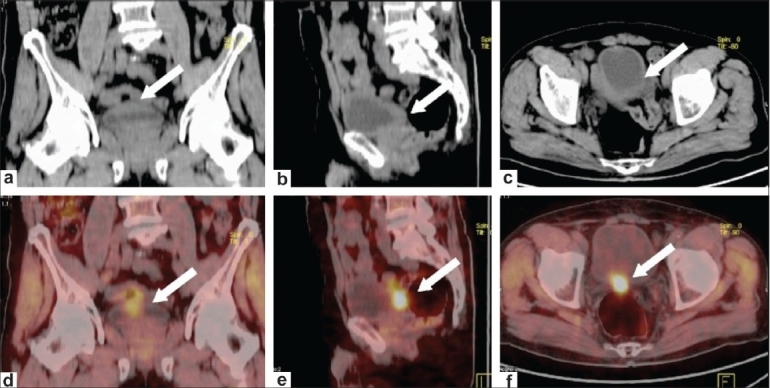
Postdiuretic delayed FDG-PET/CT acquisition in the same patient as in Figure 1. Coronal (a), sagittal (b) and axial (c) plain CT scan images reveal focal bladder wall thickening posteroinferiorly (arrows). Coronal (d), sagittal (e) and axial (f) fused PET/CT images reveal adequate tracer washout from the bladder. A focal area of hypermetabolism is noted in the thickened posteroinferior wall of the bladder (arrows), indicating active disease (recurrent cancer). Cystoscopic biopsy confirmed recurrence

**Figure 3:**
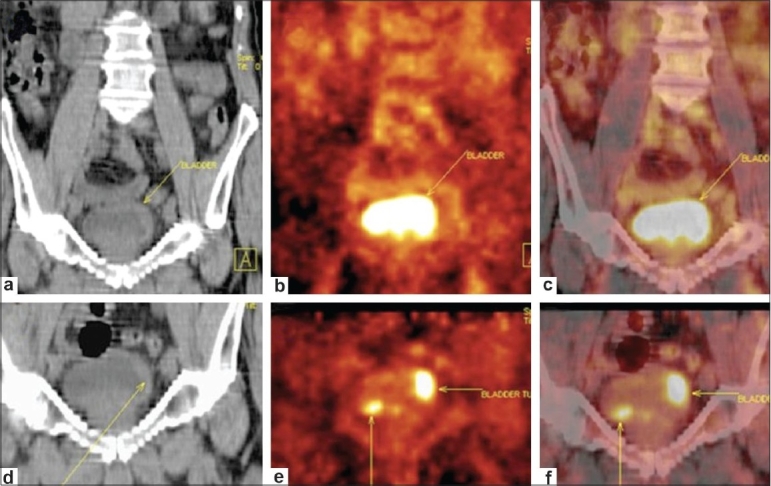
Follow-up case of invasive carcinoma of the bladder. Standard images (a-c). Coronal plain CT scan (a) image reveals focal bladder wall thickening posteriorly (arrow). Coronal PET (b) and fused PET/CT (c) images show a high concentration of tracer in the urine-filled bladder cavity. No bladder wall abnormality is discernable on PET images (arrows). Postdiuretic, delayed images (d-f). Coronal plain CT scan (d) and corresponding coronal PET (e) and fused PET/CT (f) images reveal two distinct hypermetabolic foci (arrows) in the bladder wall, indicating recurrent multifocal cancer lesions

Among the group I cases, 13 (59%) of the 22 patients with invasive TCC of the bladder and preserved bladders showed hypermetabolic bladder lesions on delayed PET/CT images, indicating active disease. In all, 16 hypermetabolic vesical lesions were detected in 13 patients, with two patients having multifocal lesions. Hypermetabolism of the bladder lesions could be determined only after washout of FDG-laden urine by furosemide injection, oral hydration and voiding [Figures 1–3]. Of the 16 hypermetabolic lesions noted in the bladder on PET images, CT detected wall thickening in the corresponding areas at only nine sites. In seven bladder wall lesions, hypermetabolism on PET images were the only abnormality detected, without any wall thickening discernable on corresponding CT images. All the 16 PET-positive lesions were confirmed to have active cancerous foci on cystoscopic biopsy. Thus, CT was false-negative for early recurrence in the bladder wall in seven of 16 lesions. There were no false PET positives found. The maximum standardized uptake value (SUV_max_) ranged from 4.5 to 12.5 for the positive bladder lesions. Histopathologic grades were grade I in four lesions, grade II in seven lesions and grade III in five lesions.

In two patients CT revealed focal wall thickening suspicious for tumor, but PET was negative and cystoscopic biopsy revealed no evidence of malignancy (i.e., a false-positive CT). Six of the 13 patients with positive PET/ CT findings were subjected to radical cystectomy and the postsurgery histopathologic examination confirmed malignant lesions (TCC) in all. The remaining seven patients were treated with bladder-preserving strategies.

Of the nine patients with negative PET/CT, seven had normal cystoscopy results and bladder wall biopsies were not performed. These cases were followed-up with 3-monthly cystoscopy and all the cases were disease-free at 1 year post treatment. In two cases with negative PET/ CT, cystoscopy and biopsy revealed early recurrent lesions of TCC (i.e., PET/CT was false-negative). The patient-based sensitivity, specificity, positive predictive value and negative predictive value for PET/CT in detecting recurrence in the bladder wall was 86.7, 100, 100 and 77.8%, respectively, and for CT, the corresponding figures were 53.8, 77.8, 77.8 and 53.8% [Table 3].

**Table 3:** Comparison of PET/CT and CT in detecting recurrent lesions in the bladder wall

Modality	Total positive (no. of patients)	True positive (no.)	False positive (no.)	True negative (no.)	False negative (no.)	Sensitivity (%)	Specificity (%)	PPV (%)	NPV (%)
PET/CT	13	13	0	07	02	86.7	100	100	77.8
CT	09	07	02	07	06	53.8	77.8	77.8	53.8

PPV - Positive predictive value; NPV - Negative predictive value

**Figure 4:**
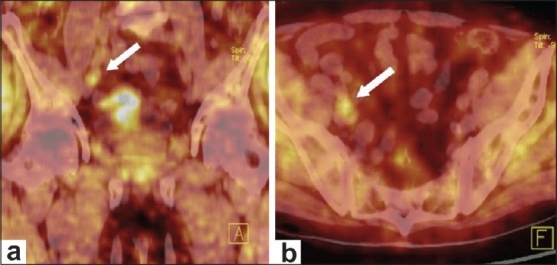
Postdiuretic delayed FDG-PET/CT acquisition in a patient of bladder cancer on follow-up. Coronal (a) and axial (b) PET/CT images reveal a hypermetabolic focus (arrows) along the common iliac vessels on the right side, indicating pelvic nodal metastasis

Pelvic nodal metastases were detected as hypermetabolic foci on PET/CT imaging in a total of six cases and confirmed on histopathology (post surgery or with needle aspiration cytology). In two of these cases, nodal lesions in the perivesical area were evident only in the delayed images and were not discernable in the early (standard acquisition) images [Figure 4]. Distant metastases were detected by PET/ CT in two patients, in both of whom the site of metastases was the skeletal system (pelvis/spine).

Analysis of the group II cases revealed that of the seven cases of TCC bladder with radical cystectomy, PET/CT was positive in only one, where a pelvic nodal uptake suggestive of metastasis was detected. CT-guided fine-needle aspiration cytology (FNAC) confirmed nodal metastasis and the patient was put on chemotherapy. In five of the seven cases, the urinary diversions showed large residual volumes and high activity within, even in delayed images. This was in spite of the furosemide injection, probably because of the incomplete evacuation of the FDG-laden urine from the conduits. However, the upper tracts were adequately cleared of FDG activity and the high activity in the diversions did not hinder the ability of PET/CT to assess the renal pelvis and ureters for metachronous lesions of TCC at these sites.

## Discussion

Accurate assessment of the extent of local recurrence as well as of distant disease (if any) is of paramount importance in accurate restaging of the disease and in planning the optimal therapy in any cancer. PET and PET/CT have been effectively used in a variety of cancers for primary staging and restaging of disease after therapy. In tumors of the urinary tract, FDG-PET has been used to accurately assess the metastatic spread but has seldom been used to image the primary/recurrent lesions in the urinary tract, primarily due to interference by high FDG concentrations in the urine.

FDG undergoes glomerular filtration similar to glucose, but it is not reabsorbed in the tubules and is largely excreted in urine, producing high activity in the urinary tract.[9] Therefore, for quite some time, FDG-PET has been considered to be of limited value in the detection of urinary tract cancers and perivesical lymph nodes.[10–13] Washing out the excreted FDG is the key to overcoming this limitation of PET imaging.

Historically, attempts have been made to improve the sensitivity of PET by achieving complete bladder washout using furosemide injection before image acquisition or by retrograde bladder irrigation; however, these techniques have met with limited success.[14,15] Investigators who used retrograde saline irrigation of the urinary bladder to remove FDG radioactivity failed to reduce tracer activity to background levels. Using retrograde saline irrigation of the bladder while imaging patients with FDG-PET, Kosuda *et al*. reported a 40% false-negative rate for the detection of recurrent or residual tumor in the bladder.[14] It was noted that continuous bladder irrigation and immediate postvoid images were not effective in reducing intravesical activity because the FDG-laden urine excreted by the kidneys kept trickling into the bladder. Cook *et al*. opine that although catheterization and drainage reduces urinary bladder activity, it still leaves small amounts of concentrated urine, which may resemble hypermetabolic lesions and be misinterpreted as such.[16] Also, bladder catheterization and retrograde filling of the bladder are invasive procedures and, in addition to increasing the risk of urinary infection in the patient, continuous bladder irrigation is known to increase radiation to the staff.[17,18]

High uptake of FDG in cancerous lesions of the bladder was first demonstrated by Harney *et al*. in rats.[19] Drieskens *et al*. used FDG-PET for preoperative staging of invasive bladder cancer and concluded that the metabolism-based information provided by FDG-PET when added to the information from CT yielded a high diagnostic and prognostic accuracy.[20] In their study, nine of 40 patients had discordance between the findings of PET/CT and that of CT alone. Of these, PET was proved to be correct in two-thirds (six of nine). They opined that, based on the presence of nodal/distant metastases detected by PET, the median survival of patients with invasive bladder cancer could be predicted. Studies have demonstrated hypermetabolism in TCC of the upper urinary tract and suggest that FDG-PET imaging may be useful in detecting primary and metastatic lesions of renal pelvic TCC as well.[21]

For follow-up and restaging of invasive bladder cancer, traditionally, a combination of cystoscopy and CT abdomen has been used. While the former determines the extent of intravesical disease, the latter is used to assess perivesical spread and detect nodal or distant metastases. It is a known fact that CT may be false-negative for early recurrence in the bladder wall as well as for metastases in normal-sized pelvic/perivesical nodes. Hence, we analyzed the utility of composite PET/CT (performed with forced diuresis and a dual-phase protocol), as compared with CT alone, for restaging of invasive bladder cancers.

The primary objective of an ideal staging modality is to achieve adequate visualization of the cancerous lesion at the primary site (the urinary tract, in the context of this study), ascertain the extent of locoregional disease and detect distant metastases if any. All these objectives were adequately achieved in our cases by the use of a dual-phase PET/CT technique, with forced diuresis and oral hydration. Initial whole-body PET/CT scan served the purpose of detecting (or ruling out) distant metastases and a postdiuretic delayed scan of the region of interest helped assess the bladder for local disease and the perivesical/pelvic region for regional nodal metastases.

In the dual-phase technique, the timing of the furosemide injection is important for obtaining satisfactory urinary dilution. Injecting a diuretic before the standard PET/CT acquisition (i.e., during the tracer uptake phase of the first 45-60 min) gives poor results as the FDG circulating in blood in high concentrations gets excreted in the urine instead of getting concentrated in the tissues. This prevents adequate dilution of urine, negating the effect of furosemide and, also, the rapid filling of the bladder leads to patient discomfort and probable patient movement during scanning. In our cases, we injected the diuretic 60-90 min after radiotracer injection and then rescanned 1-1½ h later. This technique provided excellent urinary radiotracer washout and was successful in demonstrating recurrent tumors in the bladder wall, with good lesion-to-background contrast. Bladder activity was reduced to background levels in 21 of 22 bladder-preserved patients. In patients with cystectomy and urinary diversions, adequate urinary dilution could not be obtained because increased capacity and incomplete evacuation of bladder diversions (ileal conduits) led to retention of FDG-laden urine. However, this did not appear to affect the overall assessment of the patient by FDG-PET/CT for recurrent local disease, nodal metastases or metachronous upper tract lesions.

It has been reported that iterative reconstruction algorithms provide better image quality than filtered backprojection algorithms, which can have streak artefacts.[22] In the present study, all images were constructed iteratively and no streak artefacts were observed.

Our results show that delayed FDG-PET/CT images after diuretic administration and oral hydration can demonstrate hypermetabolic lesions (representing cancer) in the urinary bladder as well as in the perivesical nodes with reasonably high clarity. Detection of locally recurrent or residual bladder tumors was significantly improved with this technique. Up to 59% of patients without cystectomy had more accurate local staging with postdiuretic dual-phase FDG-PET/CT. Delayed pelvic images after furosemide and oral hydration helped to detect pelvic lymph node metastases in two cases. These lesions would have otherwise been missed. We did not come across false-positive PET/ CT results (i.e., bladder wall uptake due to inflammatory reaction) in any of the cases who had undergone cystoscopic biopsy or transurethral resection before PET/CT. Probably the 3-month interval after the primary tumor resection allowed healing of any possible inflammatory reaction.

As a composite metabolic and anatomic diagnostic tool, PET/CT with FDG has the ability to overcome the limitations of CT and stand-alone PET.[23] We found fused PET/CT images useful for precisely locating extravesical hypermetabolic lesions. This eliminated possible false-positives that could arise from focal FDG concentration in the bowel or ureters. CT images accurately demonstrated true wall thickening in the (distended) bladder when this was present. Noncontrast imaging with a distended bladder was helpful for avoiding artifactual thickening of the walls. On the other hand, PET images accurately detected early recurrence at seven bladder sites, where the corresponding CT images did not reveal any wall thickening.

However, false-negative PET/CT results in patients with superficial recurrent lesions (confirmed on cystoscopic biopsy) were still encountered in two patients. This finding re-emphasizes the pivotal and indispensable role of cystoscopy in the follow-up of bladder cancer patients with preserved bladders. Through this study, we intend to highlight that once local recurrence in the bladder is detected on a follow-up cystoscopy, FDG-PET/CT (with additional postdiuretic images), instead of CT alone, may be the investigation of choice for restaging because it can accurately detect perivesical and distant disease as well as local recurrence in the bladder wall. PET/CT can often depict multifocality of bladder lesions and thus help the surgeon provide optimal therapy.

Our results are concordant with the findings of other studies conducted on similar lines. Anjos *et al*. have reported that PET/CT images after furosemide and oral hydration were able to overcome the difficulties posed by the urinary excretion of FDG and that detection of locally recurrent or residual bladder tumors was dramatically improved by this technique.[24] Kamel *et al*. documented improved accuracy of FDG-PET imaging in abdominopelvic malignancies when it was combined with forced diuresis.[25]

The limitation of the study is its retrospective nature. Some amount of selection bias may have been present as it is likely that only those patients of bladder cancer suspected to have recurrence were referred for PET/CT.

## Conclusion

We conclude that the technique of dual-phase PET/CT scan, along with forced diuresis and oral hydration, can aid in comprehensive assessment of recurrent lesions in invasive cancers of the urinary bladder, especially in patients with preserved bladders. The initial whole-body PET/CT scan serves the purpose of detecting (or ruling out) distant metastases and the delayed postdiuretic scan can depict the recurrent lesions in the bladder as well as metastasis in the perivesical nodes. Composite PET/CT images provide complementary structural-metabolic information and have the potential to significantly reduce the false-positives of PET and CT performed separately. Hence, in our view, whole-body FDG-PET/CT (with postdiuretic delayed imaging) should replace CT of the abdomen in the restaging protocol for recurrent invasive bladder cancers. Application of this technique may be extended to evaluation of patients with upper urinary tract cancers and other pelvic malignancies such as uterine, ovarian and colorectal cancers.
